# Mechanistic elucidation of the uncatalyzed allylation of carbonyl compounds: a combined DFT and nudged elastic band study

**DOI:** 10.1007/s00894-026-06868-4

**Published:** 2026-08-05

**Authors:** William D. A. B. Da Silva, Juliana A. B. Da Silva, Juliano C. R. Freitas, Roberta P. Dias, Júlio C. S. Da Silva

**Affiliations:** 1 Programa de Pós-Graduação Em Química, Universidade Federal Rural de Pernambuco, Recife, PE 52171-900 Brazil; 2 Núcleo Interdisciplinar de Ciências Exatas e da Natureza, NICEN, Universidade Federal de Pernambuco, Campus Do Agreste, Caruaru, PE 55014-350 Brazil; 3 Centro de Educação E Saúde, Olho D’Água da Bica, Universidade Federal de Campina Grande, Cuité, PB 58175-000 Brazil; 4 Instituto de Química e Biotecnologia, IQB, Universidade Federal de Alagoas, Campus A. C. Simões, Maceió, AL 57072-900 Brazil

**Keywords:** Carbonyl allylation, Uncatalyzed reaction mechanism, Density functional theory, Nudged elastic band, EDA-NOCV, Homoallylic alcohols

## Abstract

**Context:**

The uncatalyzed allylation of carbonyl compounds is a significant challenge in synthetic organic chemistry because of the high activation barriers often associated with non-activated substrates. This study examines the allylation of carbonyl compounds mediated by allylboronic acid to elucidate the distinct reactivity patterns observed in aldehydes and ketones. Model substrates such as methanal, propanone, and p-nitrobenzaldehyde were used to explore the full reaction pathways and related transition states. All reactions were exergonic, with activation barriers decreasing in the order propanone (35.5 kcal·mol^−1^) > methanal (23.9 kcal·mol^−1^) > p-nitrobenzaldehyde (20.8 kcal·mol^−1^), confirming that aldehydes are more reactive than ketones. NEB profiles indicate concerted but asynchronous pathways involving C–C bond formation, C–B cleavage, and proton transfer. ETS-NOCV/EDA-NOCV analysis revealed distinct electronic profiles at the transition state: methanal follows a concerted addition pathway primarily driven by σ-bond covalency, propanone proceeds via a dissociative–associative mechanism dominated by electrostatic interactions and Pauli repulsion, and p-nitrobenzaldehyde exhibits a cooperative multi-channel mechanism involving simultaneous C–C bond formation, proton transfer, and accompanying electron density redistribution within the conjugated framework. These findings clarify the electronic factors underlying the differing reactivity patterns of aldehydes and ketones in uncatalyzed allylation.

**Methods:**

All calculations were carried out using the ORCA 6.1.0 software package. The reaction pathways and transition states were studied with density functional theory (DFT) and the climbing-image nudged elastic band (CI-NEB) method. Geometry optimizations, frequency analyses, electronic structure evaluations, and NEB calculations were carried out at the ωB97X-V/def2-TZVP level of theory. Bulk solvent effects were accounted for using the SMD implicit-solvent model with water as the solvent. Single-point energy refinements were performed at the DLPNO-CCSD(T)-F12/cc-pVTZ-F12-CABS level. The chemical nature of the transition states was further analyzed using the ETS-NOCV/EDA-NOCV scheme to decompose the total interaction energy into electrostatic, Pauli repulsion, orbital, and dispersion components.

**Supplementary information:**

The online version contains supplementary material available at https://doi.org/10.1007/s00894-026-06868-4.

## Introduction

Carbonyl allylation is a pivotal transformation in organic synthesis that enables the formation of homoallylic alcohols by forming a new carbon–carbon bond between aldehydes and ketones and allyl reagents [1]. These homoallylic alcohols are versatile synthetic intermediates; their chiral forms, in particular, serve as key building blocks in the synthesis of biologically active molecules and pharmaceutical agents [1, 2]. The simultaneous introduction of a hydroxyl group and an alkene moiety in a single synthetic step further enhances the utility of this transformation [3, 4].

Traditionally, carbonyl allylation reactions are carried out under Barbier-type conditions with stoichiometric amounts of metals such as Mg, Cr, Zn, or In [1] or in the presence of transition-metal catalysts, including Zr [5], Ti [6], Co [7, 8], and Pd [9, 10]. In this context, several mechanistic pathways have been proposed for catalyzed systems, involving Lewis acid/base activation or transmetalation [11, 12].

Although there is a wide range of literature on catalytic variants, the uncatalyzed version of this reaction is still not well understood mechanistically, and a complete model of its transition states and reaction pathway has not been developed. Understanding how uncatalyzed carbonyl allylation works is challenging both theoretically and practically because there is no straightforward external activation method to control its reactivity. Unlike catalytic systems, such as those using metals, organocatalysts, or light, uncatalyzed reactions depend solely on the carbonyl’s inherent polarization, substituent electronic effects, and possibly solvent- or water-assisted proton transfers to promote C–C bond formation [1, 4, 5].

The lack of detailed structural and energetic data for transition states and intermediates in these systems limits understanding of reactivity patterns, such as the commonly observed lower reactivity of ketones relative to aldehydes [13–16]. Although aldehydes readily undergo allylboration at low temperatures, ketones typically require higher temperatures and exhibit lower compatibility with various functional groups [13]. Understanding this mechanism could provide important insights for creating milder, greener, and more user-friendly synthetic approaches.

From this perspective, this study addresses this gap by proposing a mechanistic model for the uncatalyzed allylation of carbonyl compounds using potassium allyltrifluoroborate as the allyl source. A combined computational approach employing density functional theory (DFT), nudged elastic band (NEB), and intrinsic reaction coordinate (IRC) methods was used to map the reaction pathway and identify key transition states. Three substrates were considered: formaldehyde (mAD) and acetone (prO) as simplified model systems, followed by p-nitrobenzaldehyde (p-nitroBZD) as a representative electrophile in experimental studies. Although formaldehyde is known to hydrate in aqueous solution [17], its inclusion as a model substrate is justified by its computational tractability and its frequent adoption as a minimal aldehyde model in computational studies of carbonyl reactivity and mechanism [18–21].

**Scheme 1:**
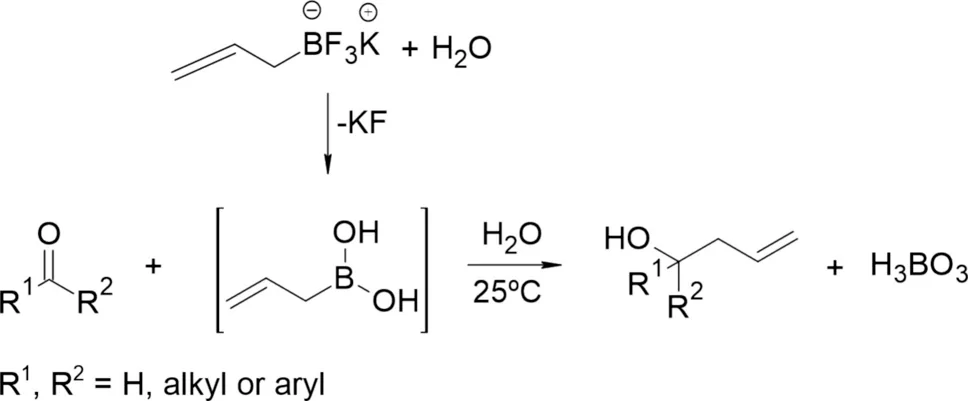
General uncatalyzed allylation of carbonyl compounds mediated by allylboronic acid in water

Based on experimental evidence [4, 7, 22–25] indicating that potassium organotrifluoroborates readily hydrolyze to their respective boronic acids, releasing potassium fluoride in aqueous media, the present study adopts allylboronic acid as the chemically relevant reactive species in water, as depicted in Scheme 1. This assumption defines the mechanistic starting point of our analysis, since the hydrolysis step precedes the C–C bond-forming event that is the primary focus of this work. Although the hydrolysis step is mechanistically relevant, a detailed computational treatment of this process falls beyond the scope of the present study, especially given its likely dependence on solvent organization and proton-transfer effects in solution. We therefore begin with the in situ–generated allylboronic acid to focus specifically on the uncatalyzed allylation step itself.

Additionally, as shown in Scheme 1, allylboronic acid reacts with carbonyl compounds to form homoallylic alcohols and boric acid. Despite the apparent simplicity of this overall transformation, some elementary events that govern its uncatalyzed course remain insufficiently characterized at the molecular level. Thus, in this work, we sought to address some of these issues by investigating these steps using DFT, CI-NEB, IRC, and EDA-NOCV analysis.

This computational study offers a plausible mechanistic explanation for key experimental findings, including the differences in reactivity between aldehydes and ketones. Additionally, the results may deepen understanding of uncatalyzed carbonyl allylation and provide useful guidance for the future development of metal-free, catalyst-free synthetic methods.

## Computational details

All quantum chemical calculations were performed using the ORCA 6.1.0 software package [26]. Geometry optimizations and electronic structure analyses were carried out using the range-separated hybrid ωB97X-V [27], a ten-parameter functional optimized through a survival-of-the-fittest strategy, which offers an efficient and accurate description of thermochemistry and noncovalent interactions. The triple-zeta valence-polarized basis set def2-TZVP was applied to all heavy atoms and hydrogen (O, C, B, H) [28].

Reaction pathways were explored using the nudged elastic band (NEB) method [29] at the ωB97X-V/def2-TZVP level of theory. Each reaction path was represented by 10 images optimized with the L-BFGS algorithm and energy-weighted spring constants (ranging from 0.01 to 0.10 *E*_h_/Bohr^2^). The reaction pathways were primarily characterized by CI-NEB, which provided the overall minimum-energy profiles and the corresponding transition-state candidates. These transition states were subsequently refined and validated by opt-ts optimization, harmonic frequency analysis, and IRC calculations [30]. The IRC results confirmed in all cases that the optimized TS structures correspond to genuine first-order saddle points connecting the adjacent minima along the proposed uncatalyzed allylation pathways.

Solvent effects were described using the SMD model for a water-implicit solvent model [31]. While the continuum approach does not explicitly represent specific solvent arrangements or hydrogen-bonding networks involved in proton transfer, it offers a straightforward method for incorporating bulk solvation effects in the comparative evaluation of substrate reactivity in C–C bond formation. Consequently, a single explicit water molecule was included in the reactive system, yielding a simple hybrid explicit/implicit solvation model. Although more comprehensive explicit-solvent models are important and may yield a more quantitative and realistic depiction of the reaction environment, such models are beyond the scope of the present study.

Thermal corrections (zero-point energy, enthalpy, and entropy) were evaluated consistently at the ωB97X-V/def2-TZVP level within the rigid-rotor harmonic-oscillator (RRHO) approximation at 298.15 K and 1 atm. All stationary points were characterized by harmonic frequency analysis: reactants and products exhibited no imaginary frequencies, whereas each transition state displayed exactly one imaginary frequency associated with the reaction coordinate.

Gibbs free energies were estimated by combining electronic energies from single-point calculations, which included the SMD solvation model for water, with thermal corrections to Gibbs free energy derived from harmonic frequency calculations at the DFT level. The reported free energies represent the sum of the SMD-corrected electronic energy and the zero-point, thermal, and entropic contributions obtained from vibrational analysis. Solvation effects were incorporated through the electronic energy term, while the thermal correction was obtained from the corresponding frequency calculation.

Single-point energy refinements were carried out at the DLPNO-CCSD(T)-F12 level [32] with the cc-pVTZ-F12-CABS auxiliary basis set [33, 34] to benchmark the reaction energetics obtained at the ωB97X-V/def2-TZVP level. For the DLPNO-CCSD(T)-F12 calculations, Gibbs free energies were estimated by combining single-point electronic energies at the coupled-cluster level, computed on DFT-optimized geometries, with thermal corrections to the Gibbs free energy derived from harmonic frequency calculations at the DFT level.

The chemical nature of the transition states was further characterized through the ETS-NOCV/EDA-NOCV scheme [35], which decomposes the total interaction energy into physically meaningful electrostatic, Pauli repulsion, orbital, and dispersion contributions.

ETS-NOCV calculations were performed as single-point evaluations on transition-state geometries previously optimized using the opt-ts algorithm. For energy decomposition, the TS structures were partitioned into two closed-shell fragments: (1) the carbonyl substrate and (2) the activated allylation complex (allylboronic acid and a water molecule). This fragmentation scheme enables the decomposition of the total interaction energy (Δ*E*_int_) into electrostatic (Δ*E*_elstat_), Pauli repulsion (Δ*E*_Pauli_), orbital (Δ*E*_orb_), and dispersion (Δ*E*_disp_) contributions. The resulting NOCV deformation densities (Δ*ρ*) provide direct visualization of charge flow associated with critical bond-forming and proton-transfer events at the transition state.

## Results and discussion

### Reaction energy and DFT validation

**Fig. 1:**
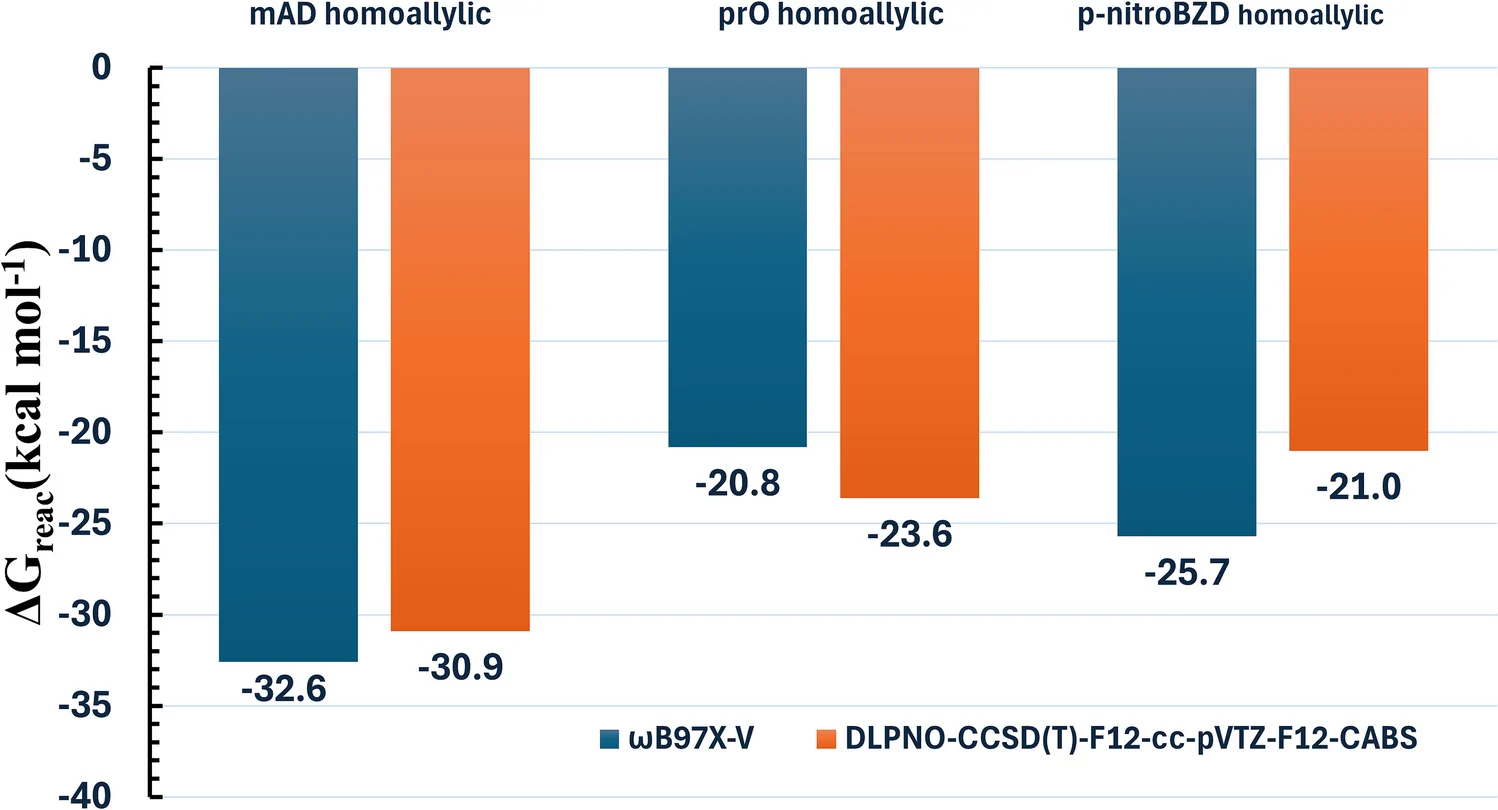
Calculated reaction Gibbs free energies for the corresponding homoallylic alcohols from methanal (mAD), propanone (prO), and p-nitrobenzaldehyde (p-nitroBZD), comparing the ωB97X-V and DLPNO-CCSD(T)-F12-cc-pVTZ-F12/CABS levels of theory

To assess the thermodynamic feasibility of the uncatalyzed allylation and to validate the computational approach, Gibbs free energy changes (ΔG) were computed using DFT with the ωB97X-V exchange-correlation functional and at the DLPNO-CCSD(T)-F12-cc-pVTZ-F12-CABS level for three model substrates: methanal (mAD), propanone (prO), and p-nitrobenzaldehyde (p-nitroBZD). The reported ΔG values (Fig. 1) represent the difference in total Gibbs free energy between the optimized products (homoallylic alcohol and boric acid) and the optimized reactants (carbonyl compound, water, and allylboronic acid).

The Gibbs free energies of reaction calculated with the ωB97X-V and DLPNO-CCSD(T)-F12 methods show consistent trends across the three substrates. As expected, aldehydes (mAD and p-nitroBZD) have more favorable reaction energies than the ketone (prO), consistent with the experimental observation that ketones often require more stringent conditions or catalytic assistance to undergo allylation efficiently [4, 23]. All methods predict exergonic reaction profiles, confirming that the allylation process is thermodynamically favorable. For the mAD system, the DLPNO-CCSD(T)-F12 reference value (−30.9 kcal·mol^−1^) closely matches the ωB97X-V value (−32.6 kcal·mol^−1^). For prO, the ωB97X-V value (−20.8 kcal·mol^−1^) is also in very good agreement with the DLPNO-CCSD(T)-F12 value (−23.6 kcal·mol^−1^).

The p-nitroBZD substrate provides the most discriminating benchmark: ωB97X-V (−25.7 kcal·mol^−1^) overestimates exergonicity by about 5.0 kcal·mol^−1^ relative to the DLPNO-CCSD(T)-F12 reference (−20.5 kcal·mol^−1^). This indicates that DFT generally over-stabilizes products in systems with strong electron-withdrawing groups. Including solvation effects via the SMD model (Figure S4 in the SI) shifts the overall thermodynamic driving force by 2.0 to 5.0 kcal·mol^−1^, depending on substrate type. This supports the experimentally observed increase in reactivity in polar, protic, and biphasic media, where water facilitates both organoboron hydrolysis and proton-transfer processes [4, 23].

Reaction energy results indicate that ωB97X-V reliably predicts the energetics of uncatalyzed allylation relative to gas-phase DLPNO-CCSD(T)-F12-cc-pVTZ-F12-CABS calculations. It closely matches the coupled-cluster reference across substrates, particularly in cases with significant electronic effects. Therefore, ωB97X-V was selected for all follow-up NEB pathway calculations and EDA-NOCV analyses, which require accurate modeling of reaction barriers, transition-state structures, and energy decomposition.

The DLPNO-CCSD(T)-F12 refinement does not produce a uniform energetic shift relative to the DFT results. Its impact varies according to the specific electronic and structural characteristics of each system. In this study, methanal, propanone, and *p*-nitrobenzaldehyde exhibit significant differences in steric demand, electronic polarization, substituent stabilization, and charge redistribution along the reaction coordinate. These factors influence the relative characterization of stationary points at both the DFT and coupled-cluster levels, thereby affecting the direction and magnitude of the resulting energy correction. Therefore, the DLPNO-CCSD(T)-F12 refinement should be considered a state- and substrate-dependent correction to the underlying DFT energetics, rather than a simple uniform offset.

Overall, the benchmarking highlights two key points: (i) the chosen DFT method consistently captures the thermodynamics of the uncatalyzed allylation pathway across most substrates and (ii) differences become more pronounced in cases with strong substituent effects, with DFT often overestimating thermodynamic stability [36–38].

### Reaction pathway: NEB and IRC calculations

#### Reaction with propanone

**Fig. 2:**
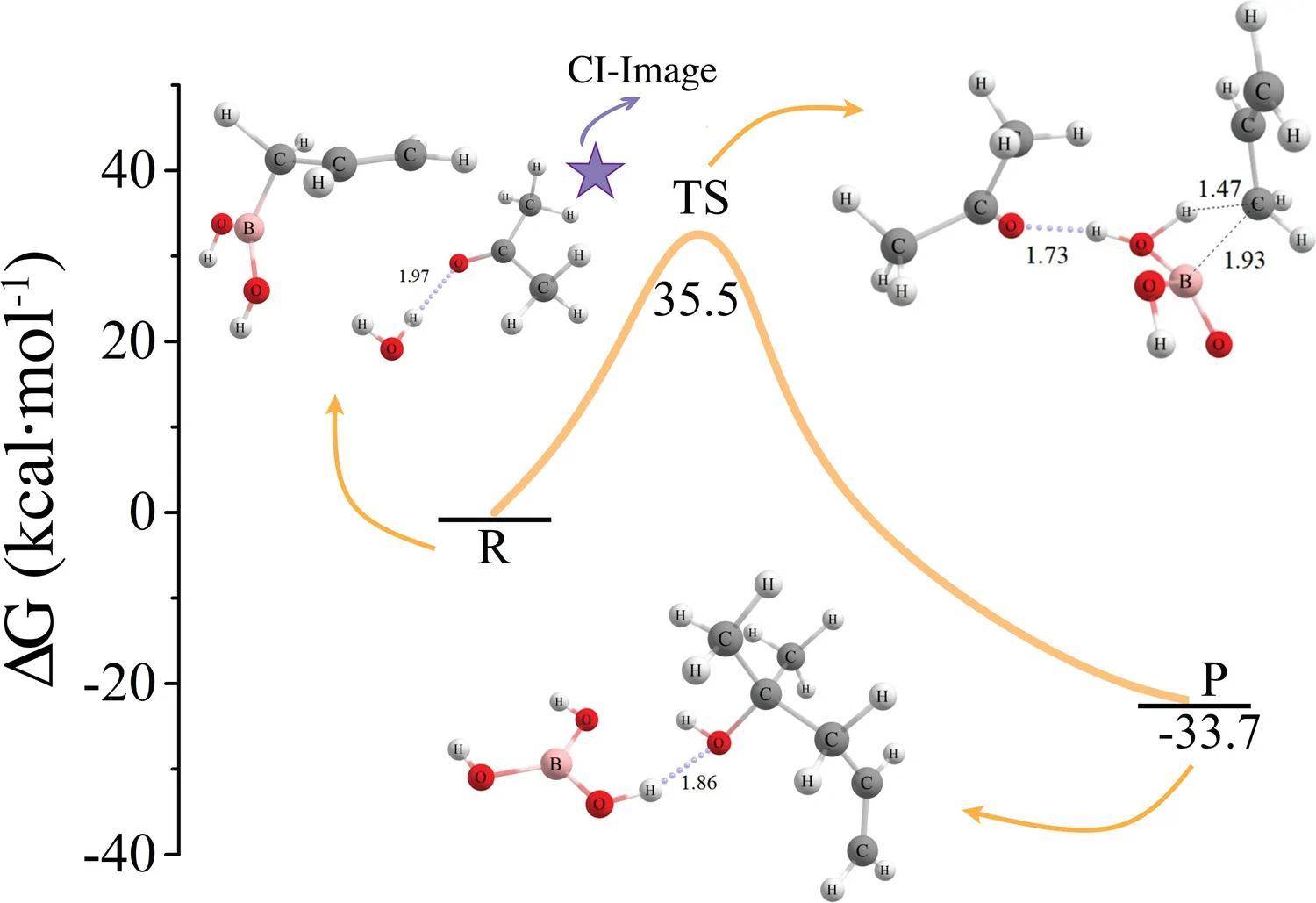
Calculated reaction energy profile for the allylation of propanone at the ωB97X-V/def2-TZVP level of theory, showing the relative energies of the reactant complex, transition state, and product complex along the proposed reaction pathway involving B–C bond cleavage and proton transfer. The CI-NEB calculation was used to generate the initial reaction path and the highest-energy image, which was subsequently refined to obtain the transition-state (TS) structure and validated by frequency and IRC analyses

In the ketone system, the reaction of propanone with allylboronic acid in the presence of a water molecule affords the corresponding homoallylic alcohol and boric acid. CI-NEB located a high-energy transition state (TS@prO, Fig. 2) with an activation free energy of 35.5 kcal·mol^−1^ and a single imaginary frequency of 1490.63i cm^−1^, primarily associated with coupled proton transfer from water and B–C bond cleavage.

The NEB-determined saddle point indicates that the uncatalyzed allylation of acetone faces a highly kinetically unfavorable pathway. In this route, water coordination to boron and partial formation of boric acid, B(OH)_3_ occur more readily than the key C–C bond formation. This can be explained by the low electrophilicity of the ketone’s carbonyl carbon when not activated by Lewis or Brønsted acids, making nucleophilic addition of the allyl fragment significantly less favorable. Ketones, due to their two alkyl groups donating electron density through inductive effects and hyperconjugation, are less electrophilic than aldehydes, raising the energy barrier for uncatalyzed allylboration [39, 40].

The computed activation free energy of 35.5 kcal·mol^−1^, although high under ambient conditions, aligns with the known low reactivity of ketones in allylation and clarifies why harsher conditions or longer reaction times are needed without catalysis [41–43]. This view matches experimental findings: Matsuoka and Kondo observed that allylation of ketones with potassium allyltrifluoroborate without a Brønsted acid (TsOH·H₂O) yielded less than 5% [12], while Nowrouzi, Thadani, and Batey demonstrated that Lewis acids like BF_3_·OEt_2_ or montmorillonite K10 improve ketone conversion [15].

#### Reaction with methanal

**Fig. 3:**
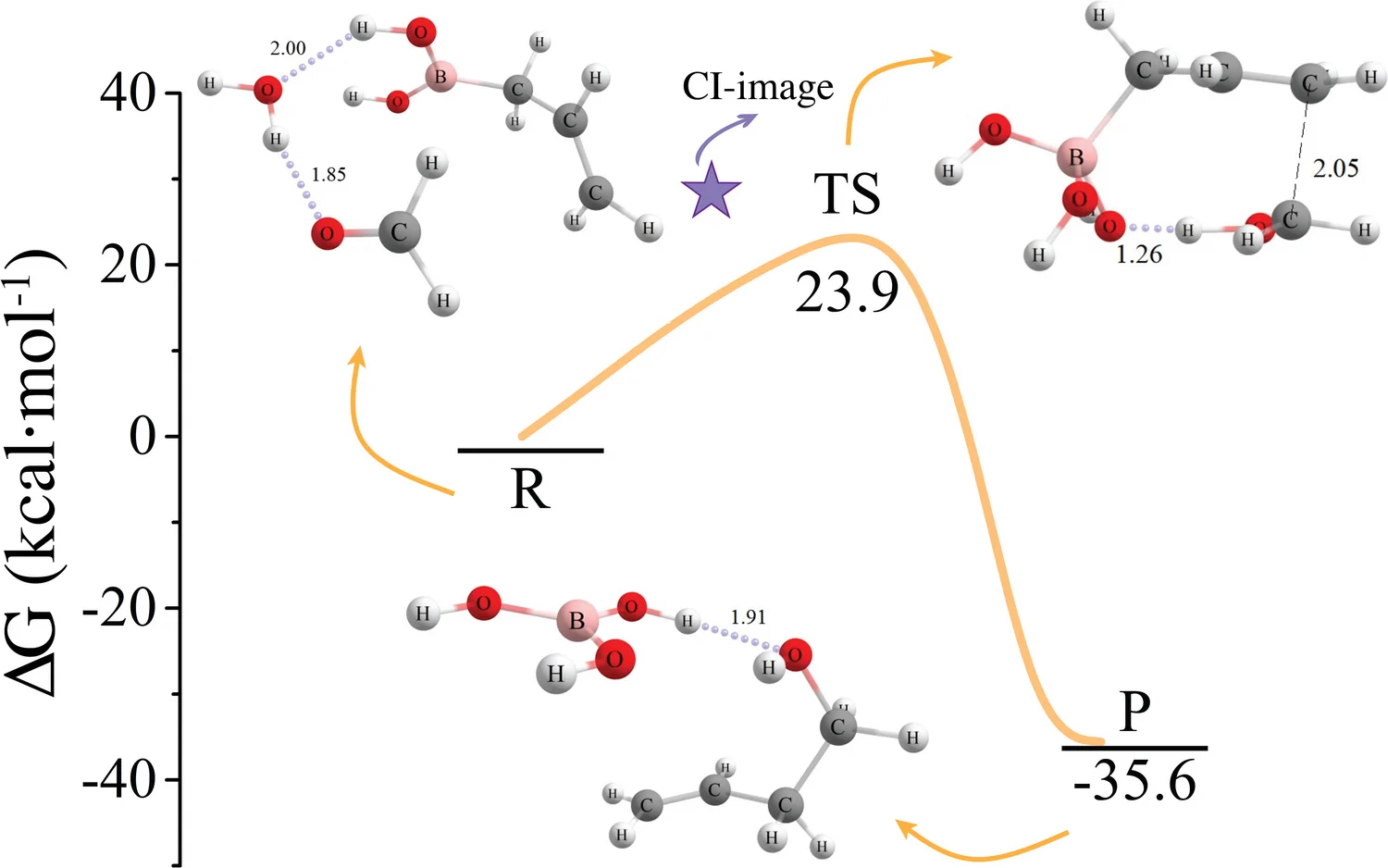
Calculated reaction energy profile for the allylation of methanal at the ωB97X-V/def2-TZVP level of theory, showing the relative energies of the reactant complex, transition state, and product complex along the proposed reaction pathway involving C–C bond formation, B–C bond cleavage, and proton transfer. The CI-NEB calculation was used to generate the initial reaction path and the highest-energy image, which was subsequently refined to obtain the transition-state (TS) structure and validated by frequency and IRC analyses

For the methanal system, CI-NEB identified a transition state (TS@mAD, Fig. 3) with a substantially lower activation barrier of about 23.9 kcal·mol^−1^ and a single imaginary frequency of 648.08i cm^−1^, consistent with the greater intrinsic electrophilicity and lower steric demand of aldehydes relative to ketones. The reaction proceeds via a concerted yet asynchronous mechanism, featuring simultaneous C–B bond breaking and C–C bond formation, coupled with proton transfer from the explicit water molecule to the carbonyl oxygen.

In the methanal system, the optimized reaction pathway exhibits a pronounced hydrogen-bonding arrangement among water, the carbonyl group, and the boronic acid moiety. This configuration aligns with methanal’s lower steric hindrance and greater electrophilicity, promoting a compact, cooperative transition state. Conversely, the propanone pathway features a more asynchronous transition-state geometry, with advanced B–C bond cleavage and less developed C–C bond formation. Consequently, a comparable hydrogen-bonding network is less favored in the propanone system.

The transition state is situated relatively late along the reaction coordinate, as reflected by the short C···C distance (2.05 Å), indicative of advanced C–C bond formation, and by the very short O(carbonyl)···H(water) distance (1.26 Å), which denotes a nearly completed proton transfer at the transition state. Collectively, these features are characteristic of a product-like, late transition state and are fully consistent with Hammond’s postulate [44] for a highly exergonic process.

#### Reaction with p-nitrobenzaldehyde

**Fig. 4:**
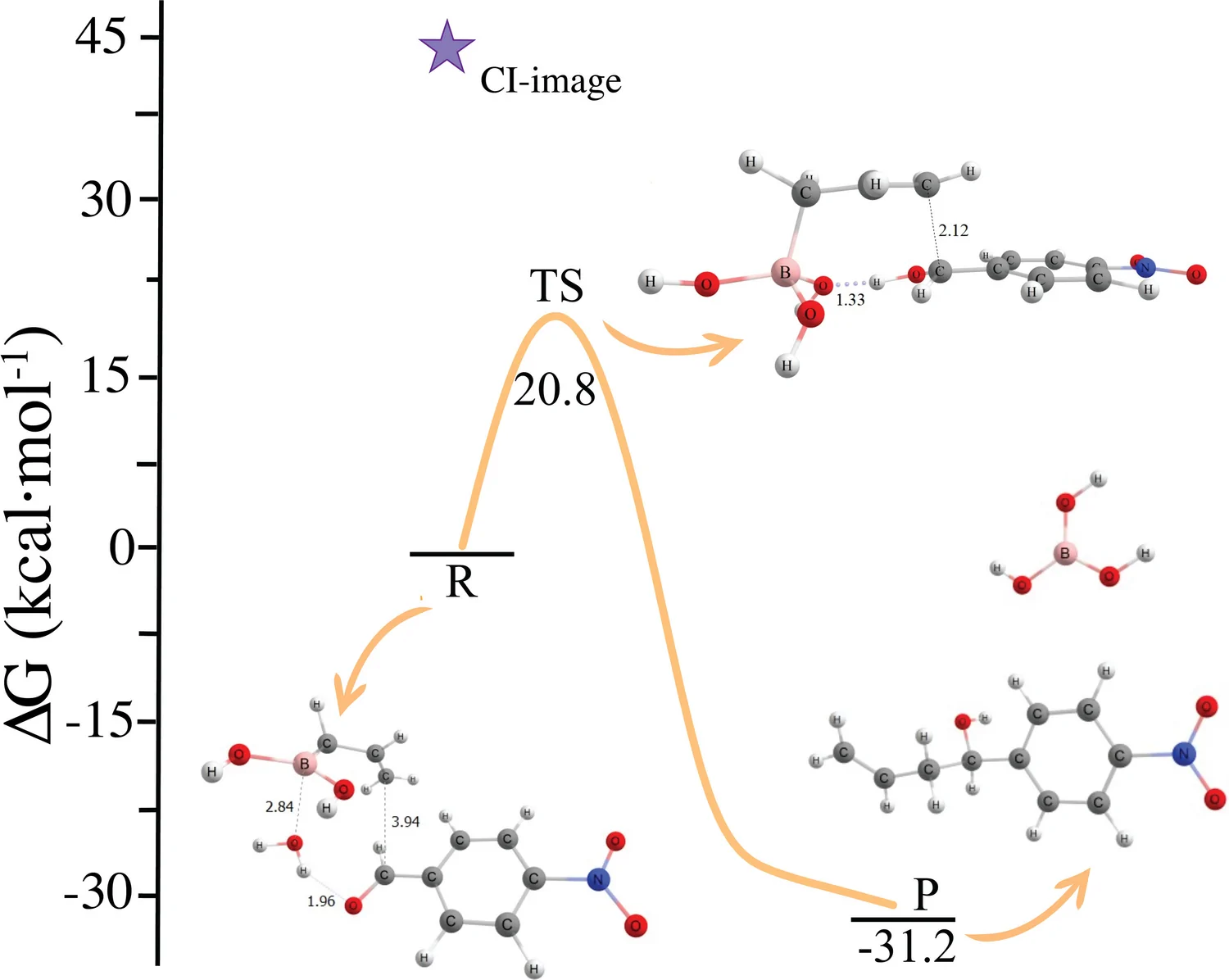
Calculated reaction energy profile for the allylation of *p*-nitrobenzaldehyde at the ωB97X-V/def2-TZVP level of theory, showing the relative energies of the reactant complex, transition state, and product complex along the proposed reaction pathway involving C–C bond formation, B–C bond cleavage, and proton transfer. The CI-NEB calculation was used to generate the initial reaction path and the highest-energy image, which was subsequently refined to obtain the transition state (TS) structure and validated by frequency and IRC analyses

The allylation of p-nitrobenzaldehyde (p-nitroBZD) proceeds with an activation barrier of 20.8 kcal·mol^−1^ (TS@p-nitroBZD, Fig. 4), and the corresponding transition state is characterized by a single imaginary frequency of 534.01i cm^−1^. This increased reactivity should result from the strongly electron-withdrawing p-nitro substituent, which enhances the electrophilicity of the carbonyl carbon, thereby promoting nucleophilic attack. The CI-NEB pathway, together with transition-state refinement and IRC validation, supports a mechanism in which proton transfer from water to the carbonyl oxygen occurs in concert with C–C bond formation. Although certain images along the discretized climbing image nudged elastic band (CI-NEB) path exhibit higher energies than the final optimized transition state (TS), these points do not represent additional maxima on the potential energy surface. Instead, they indicate the discrete nature of the NEB representation before complete transition-state refinement.

Structurally, TS@p-nitroBZD is similar to TS@mAD. The C···C distance of 2.12 Å indicates a relatively advanced stage of C–C bond formation, while the O(carbonyl)···H(water) distance of 1.33 Å shows that proton transfer is also well advanced at the transition state. The slightly longer C···C and O···H distances compared to TS@mAD suggest a somewhat earlier transition state for p-nitroBZD, despite the reaction’s greater overall exergonicity. This aligns with Hammond’s postulate [44].

**Fig. 5:**
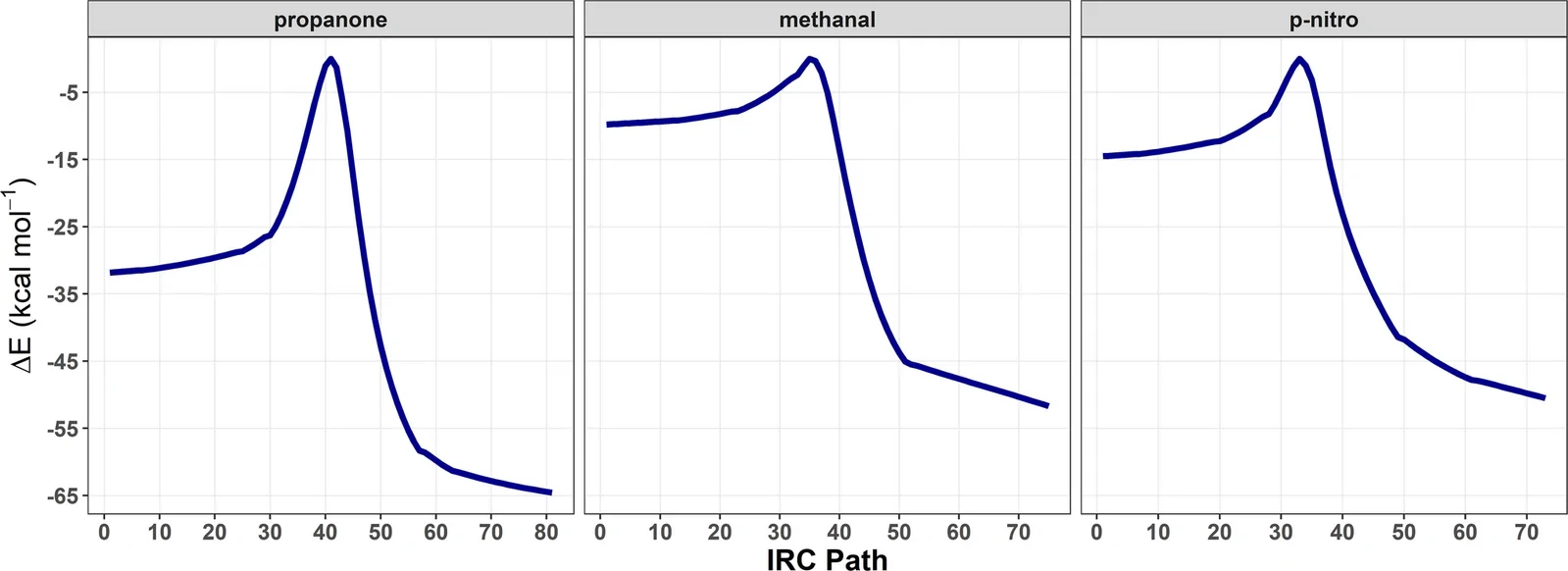
Intrinsic reaction coordinate (IRC) profiles for the transition states associated with the uncatalyzed allylation of propanone, methanal, and *p*-nitrobenzaldehyde, confirming their connectivity to the adjacent minima on the corresponding potential energy surfaces

To further confirm the transition states identified by CI-NEB, IRC calculations were performed for the propanone, methanal, and p-nitrobenzaldehyde systems (Fig. 5).

In all three cases, the IRC profiles exhibit a single maximum at the transition state, followed by a smooth downhill evolution toward the adjacent minima in both directions, with no additional maxima nor intermediate wells. This behavior confirms that the optimized structures correspond to genuine first-order saddle points and that the proposed reaction pathways connect the expected reactant- and product-side regions of the potential energy surface. The overall shape of the IRC profiles is consistent with the mechanistic picture inferred from the NEB analysis and with the exergonic character of the allylation process, as the barrier region is followed by a pronounced descent toward products for all substrates.

The lack of secondary maxima or minima in the IRC profiles suggests that the reaction follows a concerted, though asynchronous, pathway rather than proceeding through a discrete intermediate. C–C bond formation, B–C bond cleavage, and proton transfer remain strongly coupled throughout the reaction coordinate.

The complete set of NEB-derived transition-state geometries, along with their total energies and imaginary frequencies, is provided in the Supporting Information (SI1). Further validation was performed via independent opt-ts optimizations, with the corresponding reoptimized data reported in Supporting Information (SI2).

### EDA-NOCV analysis

EDA-NOCV has been widely used to elucidate the nature of chemical interactions in reactive systems [45–47]. In the present study, this method was employed to clarify the electronic factors underlying the distinct reactivity patterns of the investigated substrates, with particular emphasis on the balance between orbital and electrostatic contributions at the transition states and on how this balance relates to the contrasting behavior of aldehydes and ketones in uncatalyzed allylation.

#### TS@mAD system

**Fig. 6:**
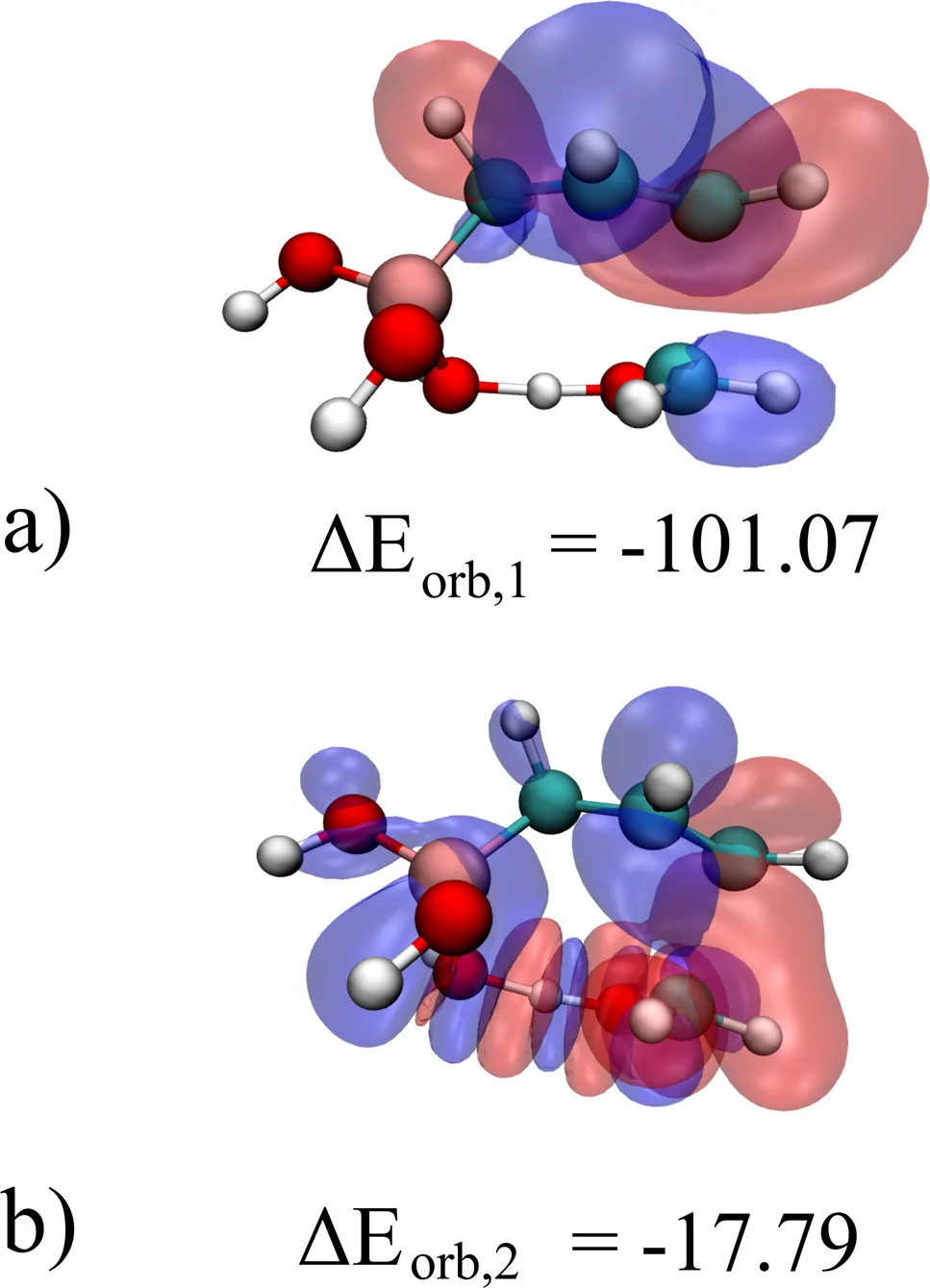
NOCV deformation densities for TS@mAD. **a** Δ*ρ*_1_ (Δ*E*_1_ = −101.07 kcal·mol^−1^) corresponds to the dominant channel associated with C–C bond formation, showing electron density accumulation between the interacting carbon atoms. **b** Δ*ρ*_2_ (Δ*E*_2_ = −17.79 kcal·mol^−1^) represents an additional redistribution of electron density within the reacting framework. Blue and red indicate regions of electron density accumulation and depletion, respectively. Atoms are colored as follows: C, blue; H, white; B, pink; and O, red

In TS@mAD, the dominant NOCV channel (Δ*E*_1_ = −101.07 kcal·mol^−1^) corresponds to the formation of the new σ C–C bond. The associated deformation density (Fig. 6a) shows significant electron accumulation between the reacting carbon atoms (blue regions) and concurrent depletion in the original orbital lobes (red regions), unambiguously confirming σ-bond formation. The large eigenvalue (~ 0.9 e^−^) indicates that bond formation is nearly complete at the transition state.

The EDA confirms this interaction is strongly covalent. The total interaction energy (Δ*E*_int_ = −95.35 kcal·mol^−1^) shows overall stabilization, with attractive forces (Δ*E*_elstat_ + Δ*E*_orb_ = −365.54 kcal·mol^−1^) greatly exceeding Pauli repulsion (Δ*E*_Pauli_ = + 334.81 kcal·mol^−1^). The kinetic–potential energy analysis (*T*_k_ = −203.48 kcal·mol^−1^; *V*_k_ = + 102.41 kcal·mol^−1^) highlights the significant charge-transfer nature of the interaction. Overall, TS@mAD features a highly polar, covalently stabilized transition state where electrostatic interactions are key to overcoming Pauli repulsion.

Secondary NOCV channels (Δ*E*_2_ = −17.79 kcal·mol^−1^ and Δ*E*_3_ = −3.08 kcal·mol^−1^) are associated with additional electron density redistribution within the developing conjugated framework, further supporting the assignment of a concerted reaction pathway.

#### TS@prO system

**Fig. 7:**
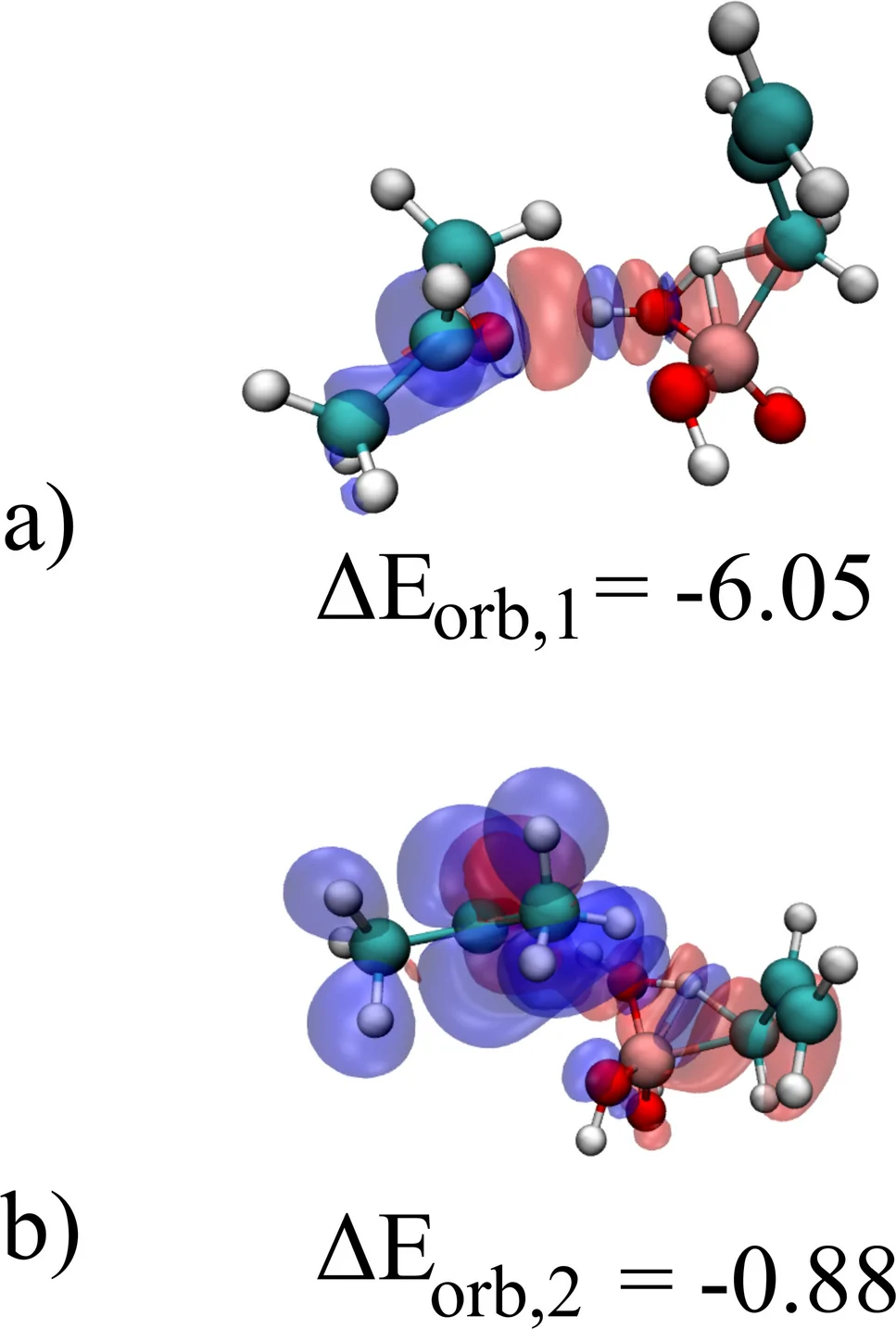
NOCV deformation densities for TS@prO. **a** Δ*ρ*_1_ (Δ*E*_1_ = −6.05 kcal·mol^−1^) corresponds to the principal interaction channel and shows electron density depletion in the C–B bonding region, accompanied by accumulation at the oxygen site. **b** Δρ_2_ (Δ*E*_2_ = −0.88 kcal·mol^−1^) represents a minor additional redistribution of electron density within the reacting framework. Blue and red indicate regions of electron density accumulation and depletion, respectively. Atoms are colored as follows: C, blue; H, white; B, pink; and O, red

In TS@prO, the main NOCV channel (Δ*E*_1_ = −6.05 kcal·mol^−1^) is much weaker than in TS@mAD, indicating the dual nature of this transition state: C–B bond breaking along with proton transfer, rather than straightforward C–C bond formation. The deformation density (Fig. 7a) reveals charge depletion from the C–B bond area and electron flow toward the oxygen, supporting a dissociative–associative mechanism. Additionally, the low eigenvalue (0.173 e^−^) suggests that charge transfer at this transition state is minimal compared to the aldehyde systems.

EDA analysis also shows that orbital interactions (Δ*E*_orb_ = −8.83 kcal·mol^−1^) provide only a slight stabilizing effect, whereas electrostatics (Δ*E*_elstat_ = −31.81 kcal·mol^−1^) are the primary contributor to the overall interaction. The secondary NOCV pair (Δ*E*_2_ = −0.88 kcal·mol^−1^, Fig. 7b) indicates minimal electronic reorganization, supporting the idea that TS@prO is dominated by electrostatic forces rather than covalent bonds.

According to the EDA-NOCV analysis, the high energy barrier for propanone is due not only to steric hindrance but also to a substantial Pauli repulsion penalty. This penalty is not adequately compensated by the weaker orbital interactions (Δ*E*_orb_) in the ketone’s less electrophilic environment. This detailed explanation clarifies why the reaction requires so much energy and provides a theoretical benchmark for the energy barrier that transition-metal catalysts must overcome to enable the reaction under milder conditions.

#### TS@p-nitroBZD system

**Fig. 8:**
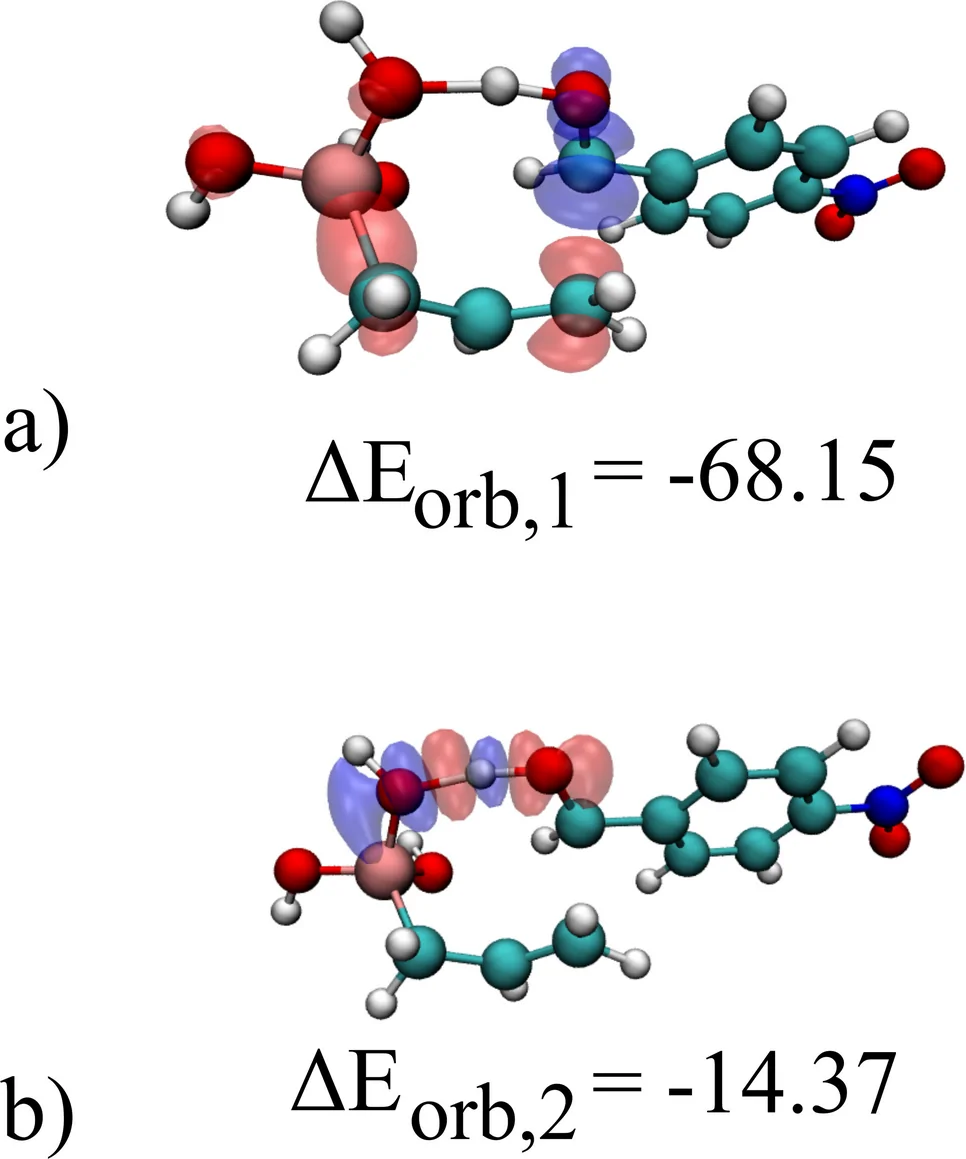
NOCV deformation densities for TS@p-nitroBZD. **a** Δ*ρ*_1_ (Δ*E*_1_ = −68.15 kcal·mol^−1^) corresponds to the principal interaction channel associated with C–C bond formation between the allylic and carbonyl carbon atoms. **b** Δ*ρ*_2_ (Δ*E*_2_ = −14.37 kcal·mol^−1^) represents an additional channel associated with proton transfer to the carbonyl oxygen. Blue and red indicate regions of electron density accumulation and depletion, respectively. Atoms are colored as follows: C, blue; H, white; B, pink; N, dark blue; and O, red

The TS@p-nitroBZD system shows the most intricate and cooperative electronic reorganization among the three substrates. Its primary NOCV channel (Δ*E*_1_ = −68.15 kcal·mol^−1^) involves C–C bond formation between allylic and aldehyde carbons. The large eigenvalue (0.881 e^−^) suggests that this new bond is nearly complete at the transition state, supported by significant charge accumulation in the emerging C–C bond area (see Fig. 8a).

The EDA results show Δ*E*_int_ as −76.75 kcal·mol^−1^, with Pauli repulsion at + 267.43 kcal·mol^−1^, which is balanced by significant attractive interactions: Δ*E*_elstat_ at −166.01 kcal·mol^−1^ and Δ*E*_orb_ at −122.16 kcal·mol^−1^. The similar sizes of the electrostatic and orbital contributions suggest that TS@p-nitroBZD features a highly polar transition state reinforced by covalent interactions, setting it apart from TS@mAD (mainly covalent) and TS@prO (primarily electrostatic).

A secondary NOCV channel (Δ*E*_2_ = −14.37 kcal·mol^−1^) involves proton transfer from the allylic fragment to the carbonyl oxygen. Despite its smaller eigenvalue (0.191 e^−^), this channel is crucial for the mechanism’s concerted nature. Additionally, a tertiary contribution (Δ*E*_3_ = −1.98 kcal·mol^−1^) appears to be associated with additional electron density redistribution within the developing conjugated framework.

The comparative EDA-NOCV analysis reveals distinct mechanistic profiles shaped by the balance between orbital and electrostatic contributions. In the mAD system, the transition state shows dominant σ-bond covalency (Δ*E*_1_ = −101.07 kcal·mol^−1^) and notable electrostatic stabilization, consistent with a concerted bond-forming mechanism. Conversely, TS@prO shows minimal orbital contribution and is stabilized primarily by electrostatic interactions, characteristic of an early, highly asynchronous transition state in which C–B bond cleavage and proton transfer are advanced, whereas C–C bond formation is not significant.

Intermediate between TS@mAD and TS@prO, TS@p-nitroBZD exhibits a balanced mechanistic profile with significant covalent C–C bond formation (Δ*E*_1_ = −68.15 kcal·mol^−1^), further stabilized by electrostatic interactions. This state also involves additional NOCV channels associated with proton transfer and electron density redistribution within the π system, indicating a more electronically complex environment. This cooperative multi-channel interaction pattern may be interpreted as a synergistic influence of the electron-withdrawing nitro group on both the thermodynamics and the electronic structure of the transition state.

Overall, the EDA-NOCV framework not only quantifies stabilizing and destabilizing contributions but also delineates the underlying mechanisms across a spectrum ranging from highly covalent and concerted (TS@mAD) through electrostatically guided and dissociative (TS@prO) to cooperative, multi-channel (TS@p-nitroBZD).

## Conclusions and final remarks

This study provides a mechanistic and electronic description of the uncatalyzed allylation of carbonyl compounds mediated by allylboronic acid. The calculated reaction profiles indicate that the process is thermodynamically favorable for all substrates examined, although the kinetic demand varies strongly with the nature of the carbonyl compound. In particular, the substantially higher barrier for propanone explains the lower reactivity of ketones under uncatalyzed conditions, whereas methanal and p-nitrobenzaldehyde display lower barriers, consistent with the greater reactivity of aldehydes.

The CI-NEB and IRC results support concerted yet asynchronous reaction pathways involving simultaneous C–C bond formation, C–B bond cleavage, and proton transfer. The EDA-NOCV analysis further demonstrates that the electronic character of the transition state varies with the substrate. For methanal, the transition state is characterized primarily by covalent σ-type C–C bond formation, consistent with a concerted addition process. In contrast, the propanone system is stabilized mainly by electrostatic interactions, with only minor orbital contributions and significant Pauli repulsion, indicating a highly asynchronous reaction pathway. *p*-nitrobenzaldehyde, in turn, displays a more cooperative transition state in which both covalent and electrostatic contributions are important, together with coupled proton transfer.

Overall, two main factors explain the lower reactivity of ketones in these uncatalyzed reactions: (1) steric hindrance, which restricts access to the carbonyl carbon and (2) electronic effects, where alkyl substituents donate electron density, decreasing the carbonyl’s electrophilicity.

The mechanistic framework developed in this work may provide a useful basis for future studies on metal-free C–C bond formation and may also help guide the rational development of milder allylation strategies that account for substrate electronic effects. Nevertheless, this study offers a mechanistic interpretation of the allylation step involved in C–C bond formation; it does not examine the complete sequence of preceding events in aqueous medium, including hydrolysis, ion pairing, and associated proton-transfer equilibria. Achieving a more comprehensive understanding of these processes will require more robust explicit-solvent models and expanded configurational sampling. Additional computational investigations in these areas could further refine the mechanistic framework and yield a more quantitative description of the overall reaction pathway.

## Supplementary information

Below is the link to the electronic supplementary material.

ESM 1 (DOCX 303 KB)

## Data Availability

The data that support the findings of this study are available from the corresponding author upon reasonable request.
