# Supplementary material for: Mechanistic elucidation of the uncatalyzed allylation of carbonyl compounds: a combined DFT and nudged elastic band study
Source: J Mol Model. 2026 Aug 5;32(9):294. doi: 10.1007/s00894-026-06868-4 (PMC13442489; doi:10.1007/s00894-026-06868-4)
Supplement: Supplementary file 1 [file 894_2026_6868_MOESM1_ESM.docx]

**Mechanistic Elucidation of the Uncatalyzed Allylation of Carbonyl Compounds: A Combined DFT and Nudged Elastic Band Study.**

William D. A. B. Da Silva,^1^ Juliana A.B. Da Silva^1,2^, Juliano C. R. Freitas^1,3^, Roberta P. Dias^4^ and Júlio C. S. Da Silva^4^

^1^Programa de Pós-Graduação em Química, Universidade Federal Rural de Pernambuco, Dois Irmãos, 52.171-900, Recife, PE, Brasil.

^2^Núcleo Interdisciplinar de Ciências Exatas e da Natureza, NICEN, Universidade Federal de Pernambuco, Campus do Agreste, 55014-350, Caruaru, PE, Brazil.

^3^Centro de Educação e Saúde, Universidade Federal de Campina Grande, Olho D'Água da Bica, 58.175-000, Cuité, PB, Brasil.

^4^Instituto de Química e Biotecnologia, IQB, Universidade Federal de Alagoas

Campus A. C. Simões, 57072-900, Maceió, AL, Brazil.

* Corresponding authors:

E-mail addresses:, [juliana.bsilva@ufpe.br](mailto:juliana.bsilva@ufpe.br) (J. A. Da Silva), roberta.dias@iqb.ufal.br (R. P. Dias), [julio.silva@iqb.ufal.br](mailto:julio.silva@iqb.ufal.br) (J. C. S. Da Silva)

Supplementary data

All calculations at the ωB97x-v /Def2-TZVP level.

Table of Contents

| **SI1** | **Cartesian coordinates, total energy and imaginary frequencies of all calculated TSs with NEB method ……………………………….** | **S2** |
| --- | --- | --- |
| **SI2** | **Cartesian coordinates, total energy and imaginary frequencies of all calculated TSs with opt-ts method ………………………………** | **S4** |
| **SI3** | **Loewdin charges for TS with ETS-NOCV/EDA-NOCV method ….** | **S6** |

# **SI1 Cartesian coordinates, total energy and imaginary frequencies of all calculated TSs with NEB method**

a) C_3_H_5_B(OH)_2_ + H_2_O + CH_2_O

Total energy: -484.88407864 E_h_

(imaginary frequency: -648.08 cm^-1^)

C	0.79485855571638	1.72446141124184	1.22289849728771

H	0.85552715737832	2.02175262892197	2.26551162506184

H	1.55831371579823	2.11537078630467	0.55704407410141

C	-0.39121778558875	1.25957391197917	0.72355444273733

H	-1.16928067553040	1.00891603596863	1.44424498553073

C	-0.70480844849921	0.93475838666814	-0.65962831985083

H	-1.69707783147583	1.33881629135362	-0.89210162384087

H	0.02496565186269	1.34371510864975	-1.36400526557052

B	-0.97441005458184	-0.70930207480184	-0.88720757525478

O	-1.41334672419868	-1.26989912494969	0.37836710024701

H	-1.57115710623641	-2.21504572515449	0.30644342992520

O	-1.83529996423551	-0.84836280987580	-2.03629676176873

H	-2.18709856165101	-1.73839450554004	-2.11706532225826

C	1.54575065989722	-0.18626237857157	1.26881210734212

H	0.66795281040285	-0.75776902211437	1.57959486383730

H	2.22367638072318	0.13189054874090	2.06241898515065

O	2.07273622816971	-0.43142943968268	0.12756032312672

H	1.31580394425556	-0.94882944956523	-0.55785530351341

O	0.36731893209287	-1.43439634638417	-1.23787790333178

H	0.51679311570064	-1.33956423318881	-2.18441235895885

b) C_3_H_5_B(OH)_2_ + H_2_O + C_3_H_6_O

Total energy: -563.51279907 E_h_

(imaginary frequency: -1490.63 cm^-1^)

C	-1.06112577896981	0.98137800327038	2.58607321923426

H	-2.10199664391705	1.28093684681825	2.65952440550077

H	-0.77714463192004	0.04569338775035	3.06108400596250

C	-0.16739745392475	1.72702115280954	1.94233936111198

H	-0.50842392721341	2.65631274987484	1.48232855888808

C	1.27408473187199	1.38124161444191	1.75318650190186

H	1.91566993147147	2.26531327004591	1.86828358736904

H	1.59591849582142	0.67060392852335	2.51770323191142

B	1.83609635895153	0.05139217005834	0.47753675843415

O	3.16089553044854	-0.30544214660477	0.74754863725122

H	3.79352842991302	0.31671358777812	0.38182785421056

O	0.88477048700309	-0.97930536267679	0.50872108969061

H	1.30269346316489	-1.79291853807091	0.80398518820686

C	-1.01414997540024	-0.80622559874990	-2.07899197588194

O	-0.06133133742401	-0.14709462100921	-2.45173412926466

C	-1.90693025594609	-0.35943894454918	-0.95343280444084

H	-2.79210037312674	0.11299946161849	-1.39699300452496

H	-2.24673013970183	-1.20926596681813	-0.35646456352845

H	-1.40009218800373	0.35702041505274	-0.30894878561309

C	-1.35700661495164	-2.10741924240283	-2.75504524314305

H	-1.14165117445911	-2.92120918062689	-2.05374172009784

H	-2.42486034221401	-2.15489095694004	-2.98742763032558

H	-0.75961194272396	-2.23769875445592	-3.65699836504711

O	1.67061278303663	1.00320211258331	-0.72951726545401

H	0.96373663827568	0.69907705442411	-1.34843575712893

H	1.32254592993807	1.47200355785487	0.28758884477722

c) C_3_H_5_B(OH)_2_ + H_2_O + C_7_H_5_NO_3_

Total energy: -920.48878468 E_h_

(imaginary frequency: -534.01 cm^-1^)

C	0.82296128014264	1.44561204760518	-0.54901119670042

H	0.65105194482668	1.86324489506906	0.43645179861871

H	1.83558254699929	1.11683875189655	-0.76542530524105

C	-0.02168186637837	1.80452391937341	-1.57214948276339

H	-0.97038356392735	2.25552628277809	-1.27853003558864

C	0.12475986420105	1.54290726705398	-2.98679017726293

H	-0.33324562273423	2.35329221595440	-3.56193223678721

H	1.15862809190878	1.38386932836388	-3.29927285213779

B	-0.79759598767006	0.18125763900512	-3.44992681698676

O	-1.96557290459821	0.16442384557659	-2.56181875922034

H	-2.62563154008573	-0.44462822214603	-2.90669186672353

O	-1.11440859181819	0.13835768044934	-4.85402945262824

H	-1.77200363903038	0.79655917763171	-5.08778526339350

C	0.05967105878825	-0.50944031555444	-0.25897508763792

O	0.83613620097090	-1.27004463139130	-0.94999617860490

H	-0.94021104276220	-0.31721379254355	-0.65135985256897

O	0.10233491312637	-1.01961747739868	-3.23541705286395

H	0.50949328633236	-1.26390261712643	-1.99440862154324

H	-0.09985939871882	-1.71318766125878	-3.86974395507001

C	0.20358127471853	-0.54752562188948	1.21959707395930

C	-0.81901745309957	-0.03066712961347	2.01442578613653

C	1.32198103616863	-1.13547534062048	1.80524639884121

C	-0.72869337008867	-0.09259131611977	3.39485221200346

H	-1.69035117243394	0.42066601145829	1.54728157864877

C	1.42417135325468	-1.20445050585918	3.18636324972891

H	2.09965828923343	-1.54696971181322	1.17153868118379

C	0.39596747274433	-0.68046846231352	3.95296338684963

H	-1.50749709367892	0.29691500549356	4.03804919200985

H	2.27837011135648	-1.65875720074295	3.67226978668399

N	0.49594628379633	-0.75713077273678	5.42661072048835

O	-0.39572285944466	-0.24982605466540	6.07367967770197

O	1.46158109790058	-1.32209723391573	5.89393465086833

# **SI2 Cartesian coordinates, total energy and imaginary frequencies of all calculated TSs with opt-ts method**

a) C_3_H_5_B(OH)_2_ + H_2_O + CH_2_O

Total energy: -484.8863832 Eh

(imaginary frequency: -669.92 cm^-1^)

C	0.74655844092309	1.73076508883367	1.20323357147837

H	0.79997645330635	2.03579167185163	2.24396891057818

H	1.48952647641255	2.14990838875491	0.53145474284527

C -0.41935073995892	1.20836365615210	0.71606181767118

H -1.17868914820493	0.92796569861100	1.44583048746735

C -0.73448467807383	0.85694454283339	-0.66111954334645

H -1.75133491070520	1.21086869254275	-0.87121421414154

H -0.04434369023710	1.30125779185344	-1.38366298345824

B -0.90092264271019	-0.81727110991101	-0.88617621505212

O -1.42148984643799	-1.34404655263000	0.36015287471576

H -1.64325238355785	-2.27287468651382	0.24401760954531

O -1.63610108490736	-1.13914401498081	-2.08937883966412

H -2.54543393569446	-0.83892212209200	-2.04203814067289

C	1.60701942917880	-0.15288861008527	1.26309609433959

H	0.75066072431559	-0.76571498524671	1.55756441348071

H	2.25114697135413	0.20256305651322	2.06864325327689

O	2.15829238973711	-0.35952320025758	0.12951825075142

H	1.41754045611214	-0.91910218259359	-0.55295036848889

O	0.47044759291503	-1.45794674540710	-1.16319945112927

H	0.58423412623305	-1.55699437922820	-2.11380227119652

b) C_3_H_5_B(OH)_2_ + H_2_O + C_3_H_6_O

Total energy: -563.5131022 E_h_

(imaginary frequency: -1488.04 cm^-1^)

C	-1.079910000	1.046883000	2.582116000

H	-2.110821000	1.381278000	2.644978000

H	-0.832884000	0.100382000	3.056364000

C	-0.154189000	1.765423000	1.953068000

H	-0.458939000	2.708259000	1.495089000

C	1.277711000	1.373586000	1.777586000

H	1.946976000	2.234455000	1.907535000

H	1.563957000	0.644624000	2.539136000

B	1.832930000	0.046929000	0.495756000

O	3.157897000	-0.309731000	0.764284000

H	3.790983000	0.321660000	0.415764000

O	0.884509000	-0.987022000	0.523333000

H	1.303977000	-1.796100000	0.829219000

C	-1.040422000	-0.772447000	-2.078393000

O	-0.090270000	-0.106337000	-2.445170000

C	-1.886887000	-0.387220000	-0.897399000

H	-2.918604000	-0.229374000	-1.230048000

H	-1.890894000	-1.201731000	-0.167787000

H	-1.506537000	0.511872000	-0.415255000

C	-1.405319000	-2.043975000	-2.800524000

H	-1.137982000	-2.888935000	-2.156209000

H	-2.482039000	-2.100261000	-2.981504000

H	-0.853212000	-2.118453000	-3.736892000

O	1.663430000	1.001500000	-0.707505000

H	0.953107000	0.706209000	-1.326534000

H	1.318000000	1.466308000	0.311786000

c) C_3_H_5_B(OH)_2_ + H_2_O + C_7_H_5_NO_3_

Total energy: -920.4932979 E_h_

(imaginary frequency: -517.87 cm^-1^)

C	0.85662840258904	1.46908973245476	-0.56686444683027

H	0.69650087272833	1.88639929506487	0.42070171279304

H	1.86627815856521	1.14066416862913	-0.79713888546207

C -0.00307741683150	1.82037725575413	-1.57954671245065

H -0.95107916162816	2.26590962080977	-1.27579171378075

C	0.12775476983721	1.55517580704964	-2.99513426999832

H -0.33943374592298	2.36296429879671	-3.56657320713216

H	1.15874872344589	1.39984815031798	-3.31888151211544

B -0.79667425651245	0.19234368863233	-3.44492058745306

O -1.95837225816220	0.18211007294851	-2.54709876878159

H -2.62087637398849	-0.42998594974295	-2.88186781332818

O -1.12718522141081	0.14473382334822	-4.84611486860935

H -1.80273304606827	0.78801625390198	-5.07010346573384

C	0.08811140725853	-0.48648151023563	-0.27165142891219

O	0.87031134471354	-1.24812706437596	-0.95612469032204

H -0.91011868151011	-0.29770216781670	-0.67227634207920

O	0.10184893248046	-1.00933479627516	-3.23413765367746

H	0.53883171338282	-1.25415979938741	-1.99445851342380

H -0.10518335355176	-1.69980221832094	-3.87020519630029

C	0.22255637382806	-0.52096041604232	1.20771819898905

C -0.80461104594496	0.00171732797521	1.99251785052719

C	1.33355733408485	-1.11239424304704	1.80449832632963

C -0.72670847144410	-0.05737150051024	3.37379156912696

H -1.66988934510292	0.45482906065511	1.51622935715942

C	1.42317114346877	-1.17880681974002	3.18674560951256

H	2.11532687141239	-1.52895474942438	1.17914139930422

C	0.39034682136693	-0.64886175283746	3.94311068927421

H -1.50976211070634	0.33646651848865	4.00908677834322

H	2.27103626776762	-1.63564740092034	3.68141562946160

N	0.47608469670411	-0.72331342021184	5.41789900859971

O -0.42317300949978	-0.21732480390183	6.05541803566362

O	1.43830319665106	-1.28544527803680	5.89554810330623

# **SI3 Loewdin charges for TS with ETS-NOCV/EDA-NOCV method**

| Atom | Charge |
| --- | --- |
| C1 | -0.201789 |
| H2 | 0.129254 |
| H3 | 0.136868 |
| C4 | -0.075096 |
| H5 | 0.161444 |
| C6 | -0.183586 |
| H7 | 0.183907 |
| H8 | 0.147829 |
| B9 | -1.249606 |
| O10 | 0.049853 |
| H11 | 0.176757 |
| O12 | 0.06054 |
| H13 | 0.1746 |
| O14 | 0.07375 |
| H15 | 0.187068 |
| C16 | -0.248201 |
| H17 | 0.126519 |
| H18 | 0.124284 |
| O19 | 0.069847 |
| H20 | 0.15576 |

Figure S1. Loewdin charges for the mAD system obtained with the ωB97X-V functional and Def2-TZVP. Carbon atoms are represented in gray, hydrogen atoms in white, and the oxygen atom in red.

| Atom | Charge |
| --- | --- |
| C1 | -0.226771 |
| H2 | 0.115283 |
| H3 | 0.118684 |
| C4 | -0.234428 |
| H5 | 0.135962 |
| C6 | -0.24765 |
| H7 | 0.134298 |
| H8 | 0.174189 |
| B9 | -1.250467 |
| O10 | 0.127237 |
| H11 | 0.198049 |
| O12 | 0.129849 |
| H13 | 0.202211 |
| O14 | 0.114046 |
| H15 | 0.184165 |
| H16 | 0.26455 |
| C17 | -0.34947 |
| O18 | 0.108554 |
| C19 | -0.256044 |
| H20 | 0.138831 |
| H21 | 0.134267 |
| H22 | 0.133892 |
| C23 | -0.253377 |
| H24 | 0.140515 |
| H25 | 0.132762 |
| H26 | 0.130865 |

Figure S2. Loewdin charges for the prO system obtained with the ωB97X-V functional and Def2-TZVP. Carbon atoms are represented in gray, hydrogen atoms in white, and the oxygen atom in red.

| Atom | Charge |
| --- | --- |
| C1 | -0.193799 |
| H2 | 0.133461 |
| H3 | 0.135476 |
| C4 | -0.075602 |
| H5 | 0.156743 |
| C6 | -0.183515 |
| H7 | 0.16557 |
| H8 | 0.154805 |
| B9 | -1.261764 |
| O10 | 0.028031 |
| H11 | 0.176291 |
| O12 | 0.066302 |
| H13 | 0.180545 |
| O14 | 0.091952 |
| H15 | 0.190862 |
| C16 | -0.193073 |
| O17 | 0.130999 |
| H18 | 0.151601 |
| H19 | 0.162782 |
| C20 | -0.137438 |
| C21 | -0.083687 |
| C22 | -0.071388 |
| C23 | -0.085041 |
| H24 | 0.157047 |
| C25 | -0.080922 |
| H26 | 0.159576 |
| C27 | -0.148298 |
| H28 | 0.16076 |
| H29 | 0.160917 |
| N30 | -0.026981 |
| O31 | -0.011937 |
| O32 | -0.010273 |

Figure S3. Loewdin charges for the p-nitroBZD system obtained with the ωB97X-V functional and Def2-TZVP. Carbon atoms are represented in gray, hydrogen atoms in white, the oxygen atoms in red and the nitrogen atom in blue.

Figure S4: Gibbs free energy of allylation reaction of methanal (mAD), propanone (prO), and *p-*nitrobenzaldehyde (*p*-nitroBZD), in gas phase (a), and in continuum solvent environment (b).
